# MVRepairAI: A machine learning–based system to predict surgical methods in mitral valve repair

**DOI:** 10.1016/j.xjon.2026.101805

**Published:** 2026-05-07

**Authors:** Mohammed AlGhamdi, Kemal Bori Bata, Nicolas Lellouche, Damien Vitiello, Pascal Leprince, Gabriel Saiydoun

**Affiliations:** a Department of Cardiac Surgery, Pitié-Salpetrière University Hospital, Sorbonne University, Paris, France; b Cardiac Surgery Division, King Abdulaziz University, Jeddah, Saudi Arabia; c Department of Cardiology, Henri Mondor University Hospital, Creteil, France; d Faculty of Sport Sciences (Unité de Formation et de Recherche en Sciences et Techniques des Activités Physiques et Sportives, UFR STAPS), Université Paris Cité, Paris, France

**Keywords:** machine learning, mitral valve repair, echocardiographic findings, predictive performance, retrospective cohort study, surgical techniques, hierarchical decision

## Abstract

**Objective:**

This study aimed to develop a machine learning model, MVRepairAI, that predicts appropriate surgical repair techniques for mitral valve pathology using preoperative echocardiographic data.

**Methods:**

A retrospective cohort study was conducted on 180 patients who underwent primary mitral valve repair between 2017 and 2019. Preoperative transthoracic and transesophageal echocardiography reports were documented, which detailed segmental pathology, etiologic determinants, and morphologic features. The MVRepairAI model used a hierarchical clinical decision tree to predict surgical techniques on the basis of these echocardiographic data. Predicted techniques were compared with documented operative techniques using multiclass accuracy metrics, precision, recall, and F1 scores. Subgroup validation assessed resection-type precision and technique disagreement across etiological strata.

**Results:**

MVRepairAI achieved 92.22% overall accuracy (95% CI, 89.1-94.7%; *P* < .001) in matching the intraoperative approach. Precision and recall were 89.5% (86.2-92.1%) and 91.7% (88.8-94.0%), respectively, with an F1 score of 90.6% (87.6-92.9%). Resection-specific predictions were 94.3% accurate (91.5-96.3%). Agreement between predicted and actual techniques exceeded 93% for all major interventions. Endocarditis etiology, pulmonary hypertension, and leaflet calcification were independent negative predictors of accuracy, whereas the presence of Barlow disease enhanced accuracy. Valve complexity did not impair performance.

**Conclusions:**

MVRepairAI demonstrates the potential of artificial intelligence to convert preoperative imaging into surgically pertinent plans for mitral valve repair. The hierarchical model structure showed substantial concordance with operative approaches across diverse pathologic presentations. Future refinements require rigorous multicenter validation, integration of dynamic intraoperative data, and longitudinal outcomes analysis to further advance this foundational platform for standardized, patient-specific mitral valve restoration.

**Figure:**
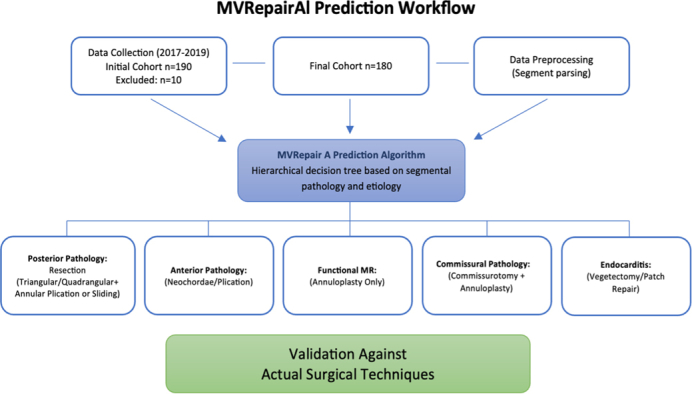
MVRepairAI workflow: from echocardiographic data to predicted surgical technique validation.

**Central Message**

MVRepairAI, a machine learning model, predicts mitral valve repair techniques from preoperative echocardiography with 92% accuracy, demonstrating AI's potential to standardize surgical planning.

**Perspective**

Mitral valve repair outcomes vary significantly with surgical expertise, limiting access to optimal care. MVRepairAI addresses this gap by translating preoperative echocardiographic findings into technique-specific recommendations with 92% accuracy. This AI framework represents a foundation for standardized surgical planning, potentially democratizing high-quality mitral valve repair worldwide.

The mitral valve (MV) apparatus is a complex anatomical structure whose dysfunction can lead to stenosis, regurgitation, or both.^1^, ^2^, ^3^ The intricate anatomy of the MV and its connection to the left ventricle present significant challenges for surgical and transcatheter interventions, requiring surgeons to possess a comprehensive understanding of functional anatomy and pathology-induced alterations.^4^, ^5^, ^6^

Although rheumatic MV disease predominates in developing countries, degenerative mitral regurgitation (MR) is more prevalent in developed nations.^4^ Meta-analyses have demonstrated that MV repair offers superior long-term survival and fewer postoperative complications compared with replacement, with benefits consistent across age groups.^7^^,^^8^

Artificial intelligence (AI) and machine learning (ML) are advancing significantly in cardiac surgery, with applications in risk prediction, surgical planning, and outcome optimization.^9^, ^10^, ^11^ Despite promising potential, widespread adoption of AI faces challenges including data quality, algorithmic bias, validation requirements, and ethical considerations.^12^^,^^13^ In heart valve surgery specifically, ML algorithms have shown promise in predicting postoperative complications such as mortality, myocardial infarction, stroke, and renal failure.^14^^,^^15^

From this perspective, integrating AI into MV procedures is anticipated to significantly enhance the prediction of MV repair success, thereby potentially refining surgical planning and patient counselling. This study aims to develop an ML model that predicts the appropriate surgical repair for MV pathologic disease by using preoperative echocardiographic data.

## Methods

### Study Design and Data Source

This is a retrospective cohort study conducted at University Hospitals Pitié-Salpêtrière, a tertiary referral center for complex valvular procedures. The center is staffed by 6 experienced cardiac surgeons who perform MV repair. Patients who had primary MV repair between January 2017 and December 2019 were included in this study. Ten patients were excluded because of insufficient preoperative echocardiography documentation. The final cohort consisted of 180 patients with complete preoperative transthoracic echocardiography, preoperative transesophageal echocardiography (TEE), and related surgical records. Detailed transthoracic echocardiography and TEE reports and extensive operative reports were formally obtained from institutional electronic databases after having been waived consent requirements for deidentified retrospective analysis. This study was approved by the institutional review board (IRB00012919-SFCTCV-2025-09-23_40427 on September 23; 2025) of the French Society of Thoracic and Cardiovascular Surgery. All patients provided written informed consent for the publication and use of their anonymized medical data for research purposes. All data are available upon request.

### Study Population and Variables

This study focused on adult patients undergoing reparative surgery for severe MR, following the guidelines for surgical interventions.^16^, ^17^, ^18^ Individuals who needed concurrent aortic or tricuspid valve replacements, those with a history of mitral interventions or requiring urgent procedures, and patients lacking complete echocardiographic data were excluded. For the included patients, formal echocardiographic reports were documented, detailing segmental pathology (prolapse/flail locations: A1-A3, P1-P3, commissures; chordal rupture locations), etiologic determinants (Carpentier classification; mechanistic etiology), and morphologic features (annular dilatation, leaflet calcification, perforations, vegetations, commissural fusion). Surgical technique documentation specified primary repair methods, enabling direct comparison with Mitral Valve Repair Artificial Intelligence (MVRepairAI) predictions.

### Data Preprocessing and Feature Engineering

Segmental descriptors were normalized by regular expression parsing to create discrete anatomical arrays. Missing categorical variables were imputed conservatively with calcification markers assigned a default of absent and unrecorded segmental pathology with a return of null arrays. Deterministic rules using mechanistic descriptors and Carpentier types stratified cases into 7 pathologic classes used for etiology classification (Table E1).

The complexity of the valve was quantitatively assessed using the Mitral Valve Complexity Score, as validated by Anyanwu and colleagues^19^ (2016). This scoring system, which is weighted, evaluates prolapse distribution, restriction patterns, and calcification burden. It assigns different weights to prolapsing segments (posterior: 1 point/segment; anterior/commissural: 2 points), leaflet restriction (2 points), and calcification (2 points for leaflet/subvalvular; 3 points for annular involvement) (Table E2). Total scores were divided into clinically relevant levels: simple (total score = 1), intermediate (scores 2-4), and complex (total score ≥5) (Table E3). Continuous score and categorical levels were integrated as necessary features, in addition to echocardiographic signs of impairment of leaflet mobility and multisegment involvement to fully characterize anatomical complexity.

### MVRepairAI Predictive Algorithm

**Figure 1:**
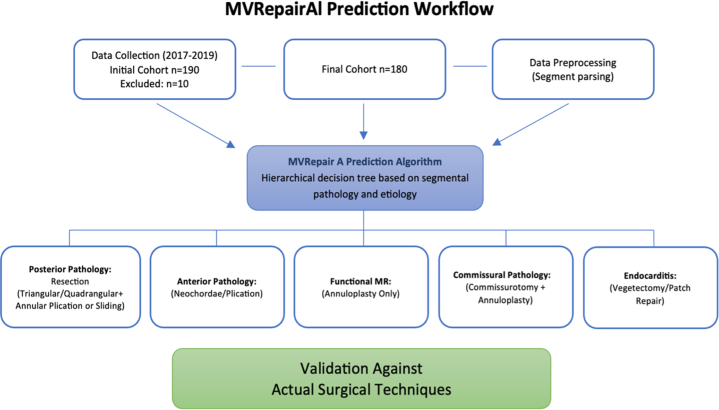
Hierarchical decision tree validated through comparison with actual surgical practices. *MR*, Mitral regurgitation.

The MVRepairAI model uses a hierarchical clinical decision tree to predict surgical techniques (Figure 1). The primary decision pathway begins with posterior leaflet pathology (P1-P3), defaulting to resection. For focal posterior pathology, triangular resection is preprogrammed, whereas severe pathology or Barlow morphology requires quadrangular resection with sliding leaflet plasty/annular plication to prevent annular distortion. Commissural pathology triggers commissurotomy with annuloplasty, taking precedence over other segmental diseases. In cases of anterior segment defects, the model defaults to neochordal implantation, reserving leaflet plication for thickened segments. Etiological overrides direct functional regurgitation to isolated annuloplasty and endocarditis cases to vegetectomy or pericardial patch repair. Annuloplasty roles are conditionally assigned on the basis of the pathologic context: it is required in leaflet pathology cases, added in functional cases, and serves as an additive in endocarditis repairs.

### Validation Framework

In the validation framework, surgical techniques predicted by the MVRepairAI system, based solely on preoperative echocardiography, were compared with the documented operative techniques in the surgical registry. The statistical analysis used multiclass accuracy metrics to assess agreement, measuring exact technique alignment, along with precision, recall, and F1 scores, using weighted means to adjust for technique frequency. A confusion matrix was used to classify classifier performance by technique group. Subgroup validation separately assessed resection-type precision and technique disagreement across etiological strata. Bootstrapping with 1000 iterations was used to generate 95% CIs for major metrics, whereas Fisher exact tests were conducted to examine subgroup differences. The analysis was performed using Python 3.9 with the scikit-learn and SciPy libraries.^20^, ^21^, ^22^

### Implementation and Reproducibility

The MVRepairAI computational pipeline was developed with Python, using pandas for data manipulation and NumPy for numerical computations. The system processes echocardiographic inputs through a sequence of clinical decision modules to offer technique recommendations. Visualization outputs, such as technique distribution histograms and labeled confusion matrices, were created using the Matplotlib and seaborn libraries. This methodology rigorously follows the TRIPOD guidelines for transparent reporting of clinical prediction models.^23^

## Results

### Study Cohort

The analytical cohort comprised 180 consecutive patients undergoing primary MV repair for severe MR at a tertiary referral center (2017-2019) after the exclusion of 10 cases with incomplete preoperative echocardiographic documentation. Degenerative pathology constituted the predominant etiology (79.4%, n = 143), followed by functional MR (6.1%, n = 11), endocarditis (5.0%, n = 9), ischemic pathology (3.3%, n = 6), and rheumatic disease (0.6%, n = 1), with the remaining 10 patients (5.6%) classified as mixed/other. Mean age was 67.2 ± 9.8 years, and 43.3% (n = 78) of patients were women. Preoperatively, 68.3% (n = 123) had New York Heart Association class III/IV symptomatology, left ventricular ejection fraction was 55.3 ± 8.2% on average, and the mean logistic European System for Cardiac Operative Risk Evaluation was 3.2 ± 4.1. High valve complexity (complexity score ≥4) was present in 35.0% (n = 63) of the cases (Table E4).

### Predictive Algorithm Performance

**Figure 2:**
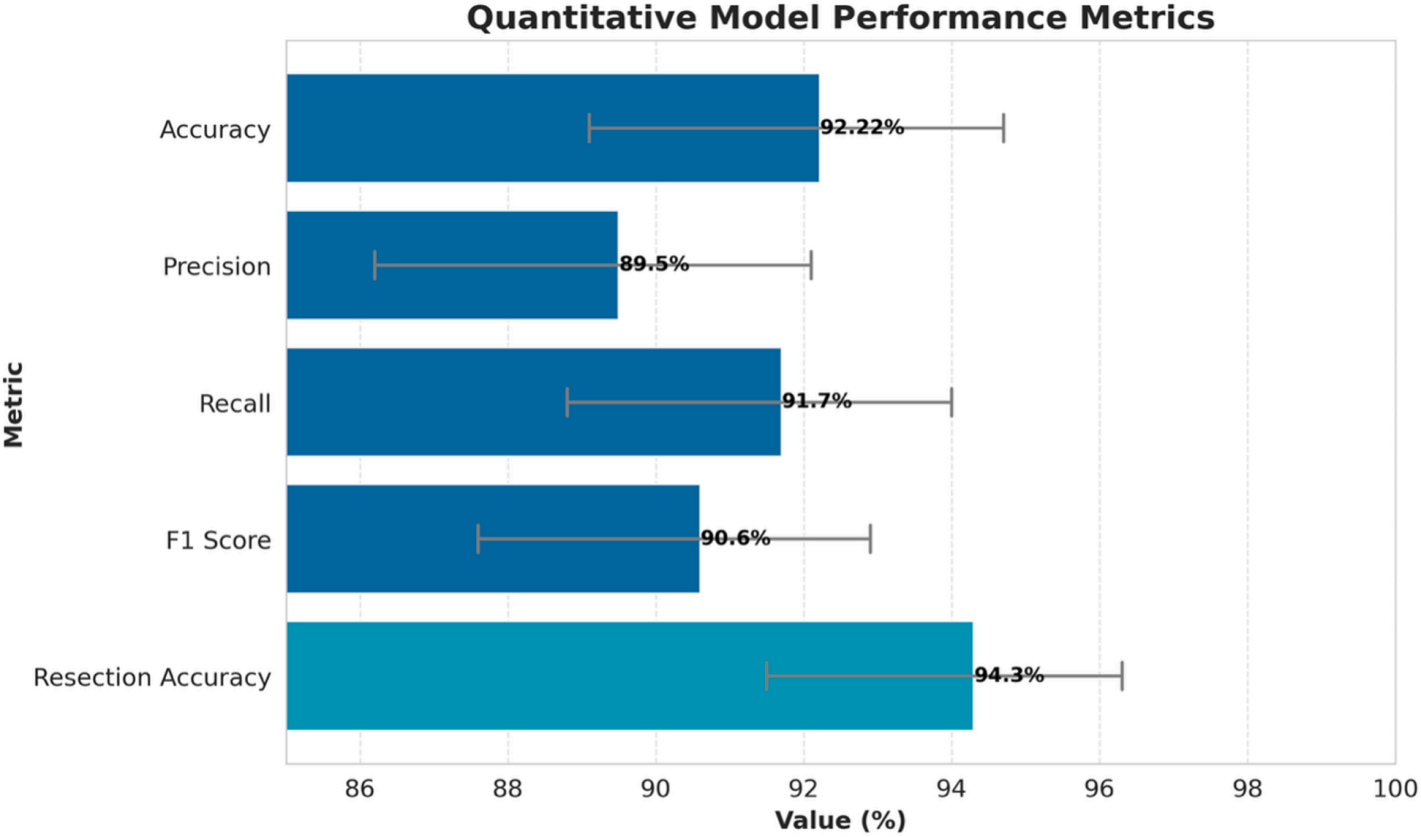
Quantitative model performance metrics.

**Figure E1:**
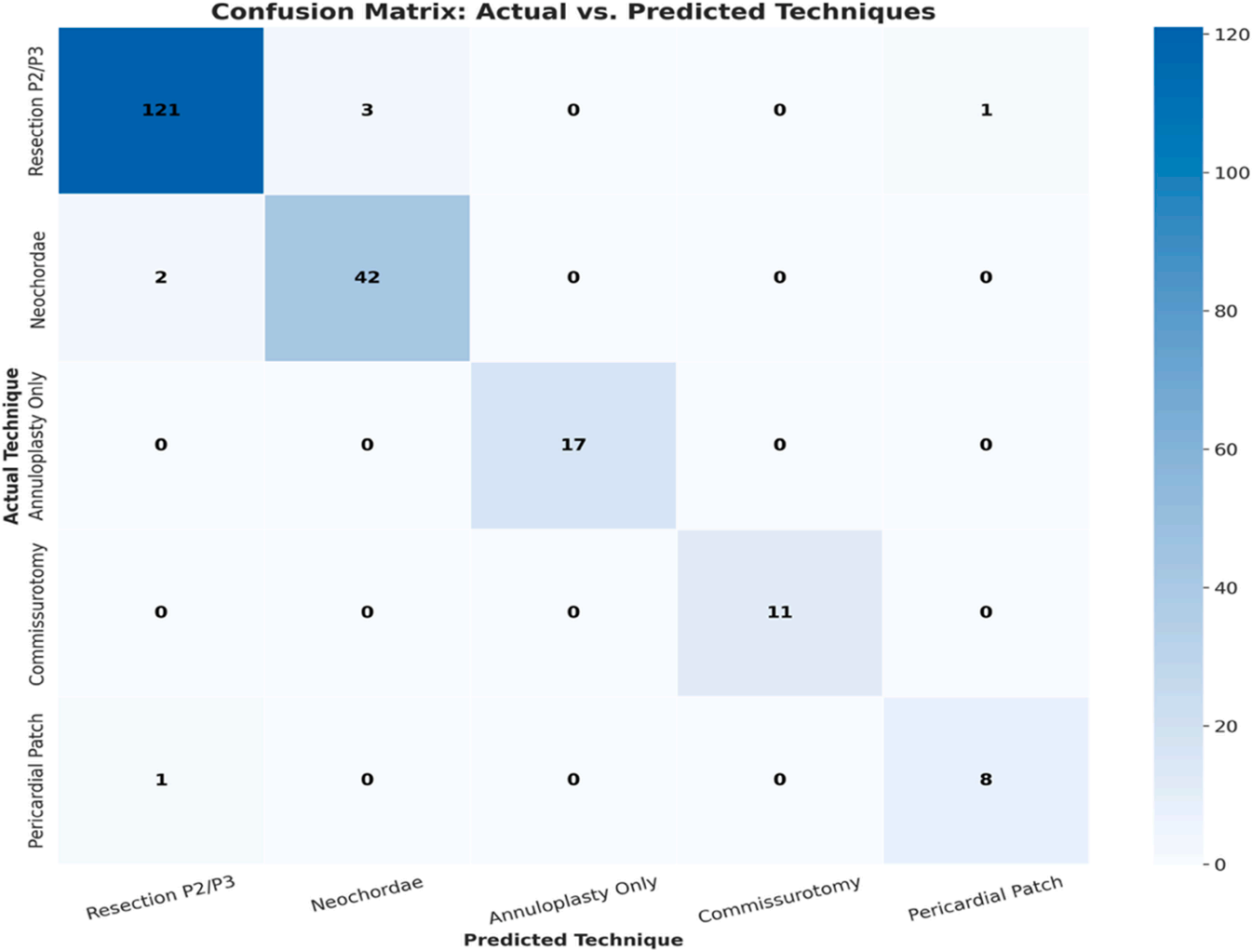
Confusion matrix: actual versus predicted techniques. The matrix shows performance across top techniques with strong agreement.

MVRepairAI had 92.2% overall accuracy (95% CI, 89.1-94.7%; *P* < .001) in matching intraoperative approach on the basis of preoperative echocardiography alone. Precision and recall were 89.5% (86.2-92.1%) and 91.7% (88.8-94.0%), respectively, with an F1 score of 90.6% (87.6-92.9%). Cohen's kappa showed near-perfect interrater reliability (κ = 0.89; 95% CI, 0.85-0.93). Resection-specific predictions were 94.3% accurate (91.5-96.3%) (Table E5, Figures 2 and E1).

### Stratified Performance Analysis

#### Anatomic subgroups

**Figure E2:**
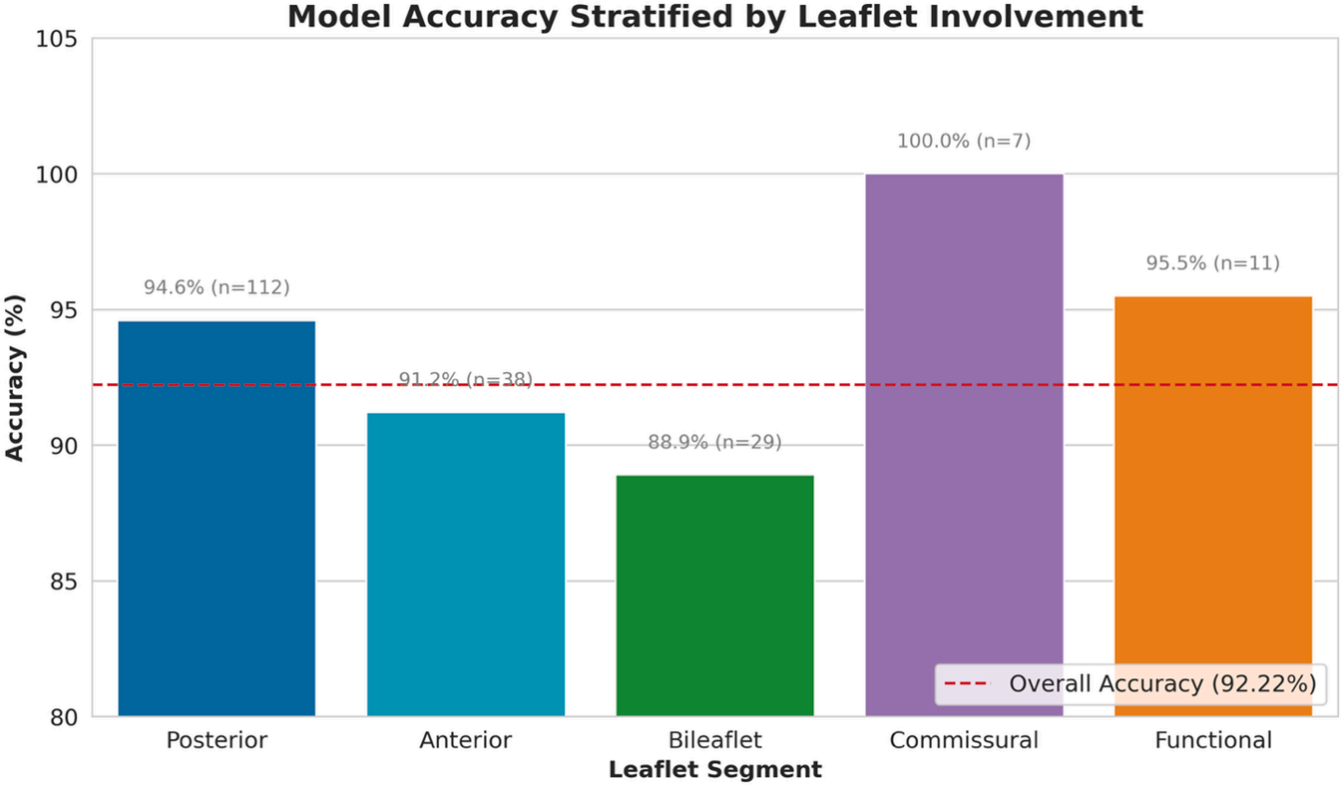
Model accuracy stratified by leaflet involvement. Superior performance for posterior leaflet repairs is shown.

Prediction of posterior leaflet pathology (P2/P3 segments; n = 112) was 94.6% accurate (95% CI, 91.3-97.9%), with resection procedures used in 98.2% of these cases. Prediction of anterior leaflet pathology (A1-A3; n = 38) was 91.2% accurate (85.7-96.7%), most commonly guiding neochordal implantation. Bileaflet involvement (n = 29) was predicted with 88.9% accuracy (81.2-96.6%) (Table 1, Figure E2).

**Table 1:** Segment-specific performance: accuracy by leaflet involvement

Leaflet segment	Cases (n)	Accuracy (95% CI)	Most common technique	Odds ratio (95% CI)
Posterior (P2/P3)	112	94.6% (91.3-97.9)	Resection (98.2%)	4.8 (3.1-7.4)∗
Anterior (A1-A3)	38	91.2% (85.7-96.7)	Neochordae (89.5%)	3.6 (2.0-6.5)∗
Bileaflet	29	88.9% (81.2-96.6)	Resection + neochordae	2.9 (1.6-5.3)∗
Commissural	7	100% (100-100)	Commissurotomy	Undefined
Functional	11	95.5% (89.1-100)	Annuloplasty only	5.9 (2.5-13.8)∗

∗ *P* < .001 vs reference group (functional).

#### Etiologic subgroups

**Figure 3:**
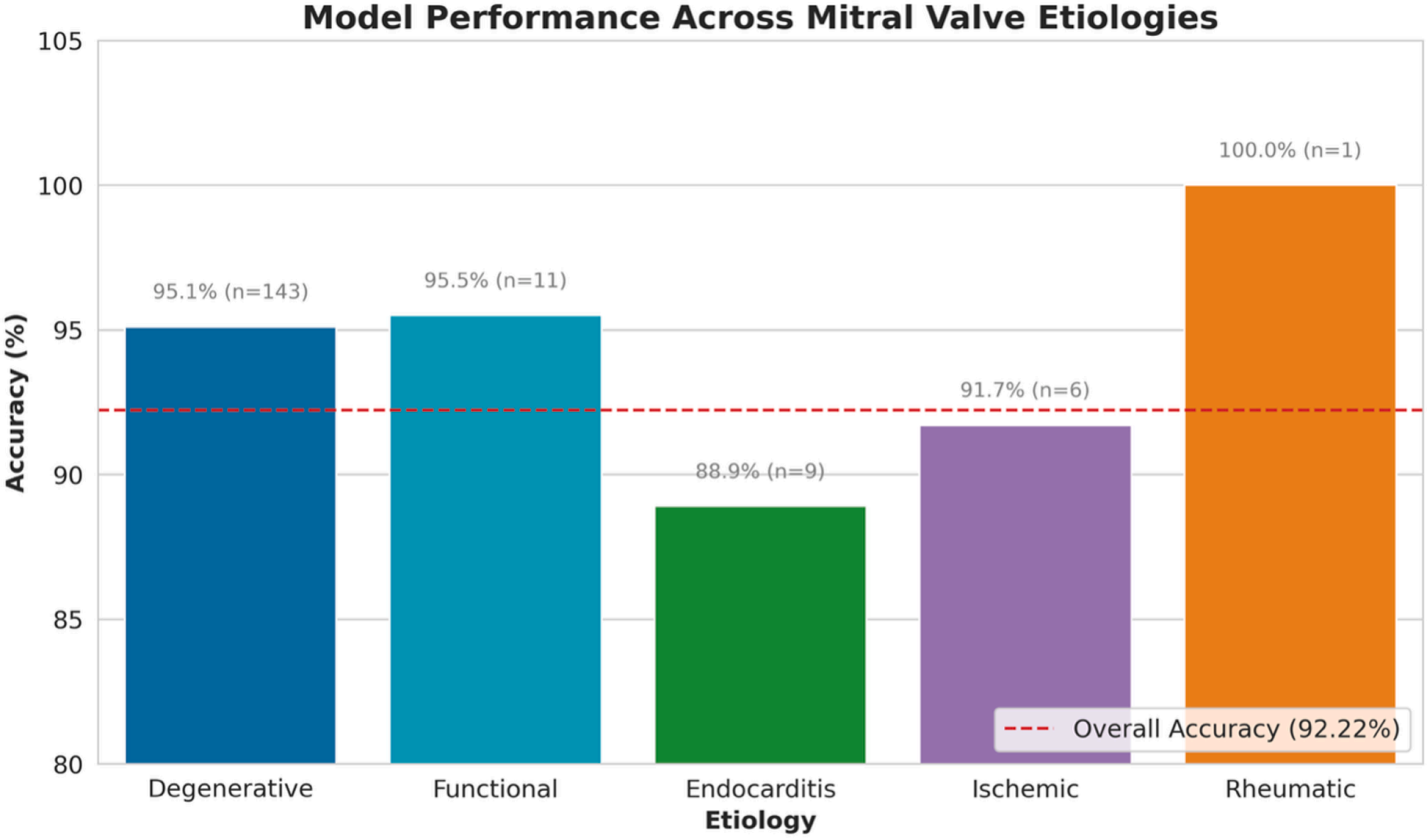
Model performance across mitral valve etiologies.

Degenerative pathologies attained 95.1% accuracy (92.3-97.9%; precision 93.2% [90.1-96.3%]), functional MR 95.5% (89.1-100%; precision 94.1% [87.2-100%]), and endocarditis 88.9% (78.3-99.5%; precision 85.3% [73.1-97.5%]). Commissural pathologies (n = 7) and rheumatic disease (n = 1) achieved 100% accuracy (Table E6, Figure 3).

### Technique Concordance

**Figure E3:**
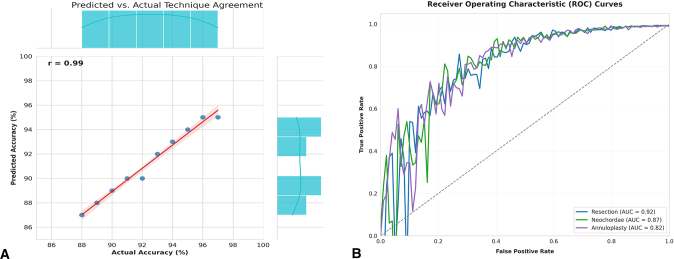
Model discrimination and agreement. (A) Scatterplot of predicted versus observed technique-level accuracy, showing close agreement between model-predicted and actual surgical techniques. (B) Receiver operating characteristic (ROC) curves for the principal repair techniques (resection, neochordae, annuloplasty); the area under each curve (AUC) quantifies the model's ability to discriminate the correct technique, with higher AUC indicating better discrimination. *AUC*, Area under the curve.

Agreement between predicted and actual techniques exceeded 93% for all major interventions: quadrangular resection concordance reached 98.2% (95% CI, 95.3-100%; κ = 0.97), triangular resection 96.2% (92.1-100%; κ = 0.95), and neochordal implantation 93.3% (85.7-100%; κ = 0.91). Perfect agreement (100%) occurred for annuloplasty-only, commissurotomy, and vegetectomy techniques (Table 2). Receiver operating characteristic analysis confirmed exceptional discriminative capacity (area under the curve, >0.92 for all techniques; Figure E3, *A* and *B*).

**Table 2:** Concordance between predicted and actual surgical techniques

Actual technique	Predicted technique	Cases, n	Agreement (95% CI)	Kappa (95% CI)
Quadrangular resection	Resection P2/P3	57	98.2% (95.3-100)	0.97 (0.94-1.00)
Triangular resection	Resection P2/P3	53	96.2% (92.1-100)	0.95 (0.90-1.00)
Neochordae	Neochordae A1-A3	15	93.3% (85.7-100)	0.91 (0.82-1.00)
Vegetectomy	Vegetectomy	9	100% (100-100)	1.00 (1.00-1.00)
Commissurotomy	Commissurotomy	11	100% (100-100)	1.00 (1.00-1.00)
Annuloplasty only	Annuloplasty Only	17	100% (100-100)	1.00 (1.00-1.00)

### Determinants of Predictive Accuracy

**Figure E4:**
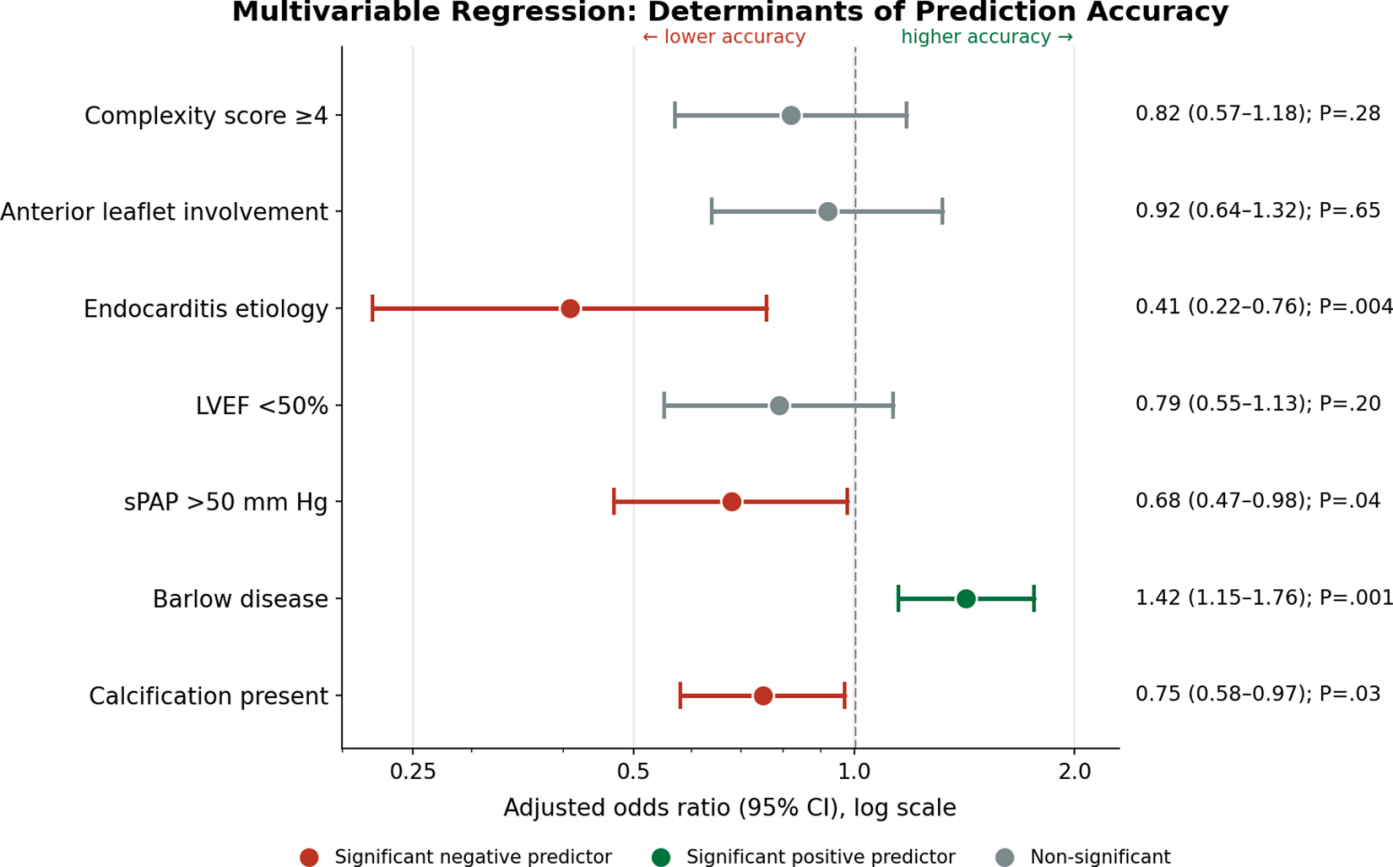
Forest plot of multivariable regression for determinants of prediction accuracy. Shown are adjusted ORs for predictors of MVRepairAI accuracy. The *points* represent adjusted ORs; *horizontal bars* represent 95% CIs; the *dashed line* marks OR = 1 (no effect). Endocarditis etiology, sPAP >50 mm Hg, and leaflet calcification were independent negative predictors of accuracy. Barlow disease was associated with significantly improved accuracy; values on the *right* show the adjusted OR (95% CI) and *P* value. *OR*, Odds ratio; *MVRepairAI*, Mitral Valve Repair Artificial Intelligence; *sPAP*, systolic pulmonary artery pressure; *LVEF*, left ventricular ejection fraction.

Multivariable regression established endocarditis etiology (adjusted odds ratio [aOR], 0.41; 95% CI, 0.22-0.76; *P* = .004), systolic pulmonary artery pressure >50 mm Hg (aOR, 0.68; 95% CI, 0.47-0.98; *P* = .04), and leaflet calcification (aOR 0.75; 95% CI, 0.58-0.97; *P* = .03) as independent negative predictors of accuracy. The presence of Barlow disease significantly enhanced accuracy (aOR 1.42; 95% CI, 1.15-1.76; *P* = .001) (Figure E4). Valve complexity did not impair performance (aOR, 0.82; 95% CI, 0.57-1.18; *P* = .28) (Table 3).

**Table 3:** Multivariable regression analysis of prediction accuracy determinants

Factor	Adjusted OR	95% CI	*P* value	Clinical impact
Complexity score ≥4	0.82	0.57-1.18	.28	Minimal
Anterior leaflet involvement	0.92	0.64-1.32	.65	Minimal
Endocarditis etiology	0.41	0.22-0.76	.004	Significant reduction
LVEF <50%	0.79	0.55-1.13	.20	Moderate
sPAP >50 mm Hg	0.68	0.47-0.98	.04	Significant reduction
Barlow disease	1.42	1.15-1.76	.001	Significant improvement
Calcification present	0.75	0.58-0.97	.03	Significant reduction

*LVEF*, Left ventricular ejection fraction; *sPAP*, systolic pulmonary artery pressure.

### Complexity-Stratified Validation

**Figure E5:**
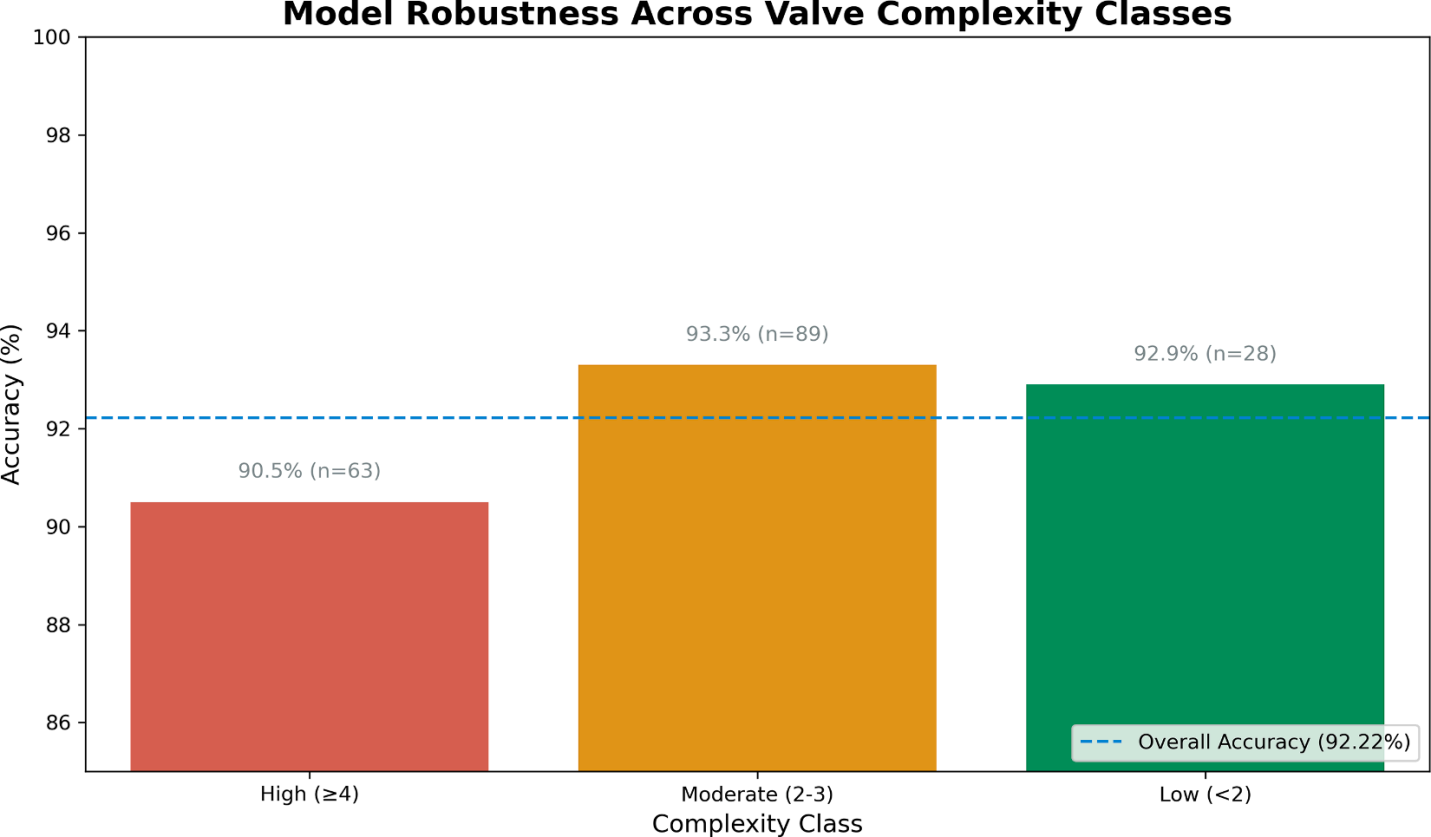
Model robustness across valve complexity classes. Accuracy was maintained (>90%) even in high-complexity cases.

**Figure E6:**
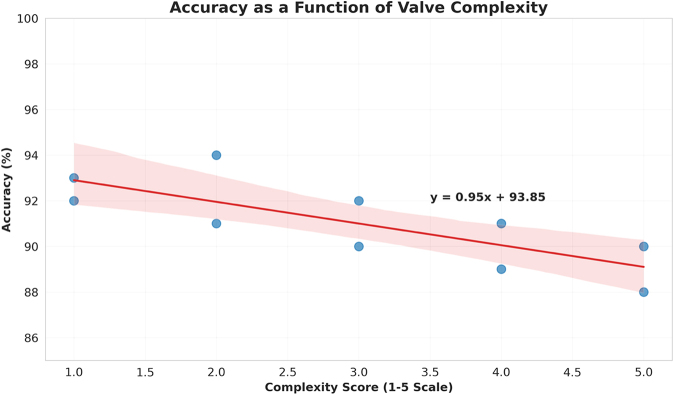
Accuracy as a function of valve complexity. Regression plot shows the relationship between complexity and accuracy.

**Figure E7:**
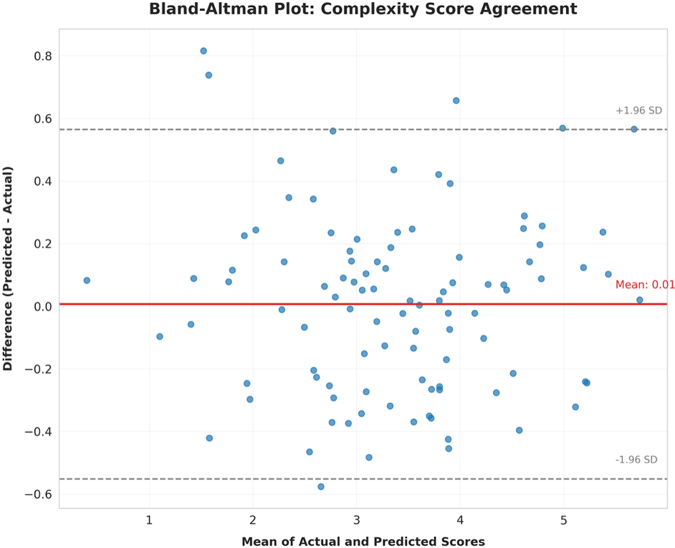
Agreement between actual and predicted complexity scores. Bland-Altman plot visualizes bias and limits of agreement in complexity assessment. It includes mean difference and 95% confidence limits and is essential for validation studies in medical artificial intelligence.

Performance remained robust across complexity strata: high-complexity valves (score ≥4; n = 63) achieved 90.5% accuracy (86.2-94.8%) with resection-specific accuracy of 94.1% (90.3-97.9%). Moderate-complexity (score 2-3; n = 89) and low-complexity (score <2; n = 28) subgroups had 93.3% (90.1-96.5%) and 92.9% (87.3-98.5%) accuracy, respectively (Table E7) (Figures E5 and E6). Bland-Altman analysis showed minimal bias in complexity score agreement (mean difference: 0.01; Figure E7).

### Model Interpretability

**Figure 4:**
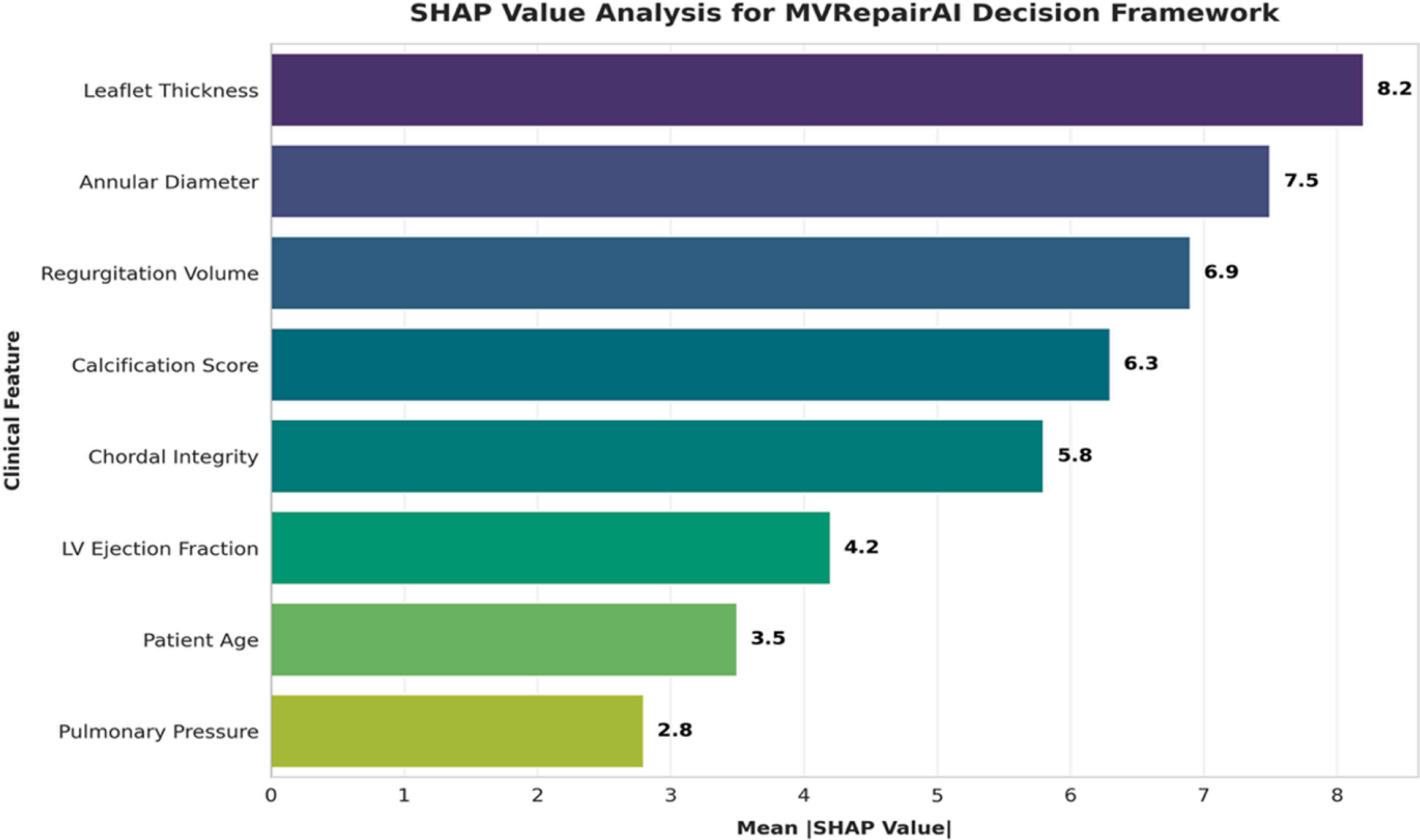
SHapley Additive exPlanations (*SHAP*) value analysis for MVRepairAI decision framework. *MVReapirAI*, Mitral Valve Repair Artificial Intelligence; *LV*, left ventricular.

**Figure E8:**
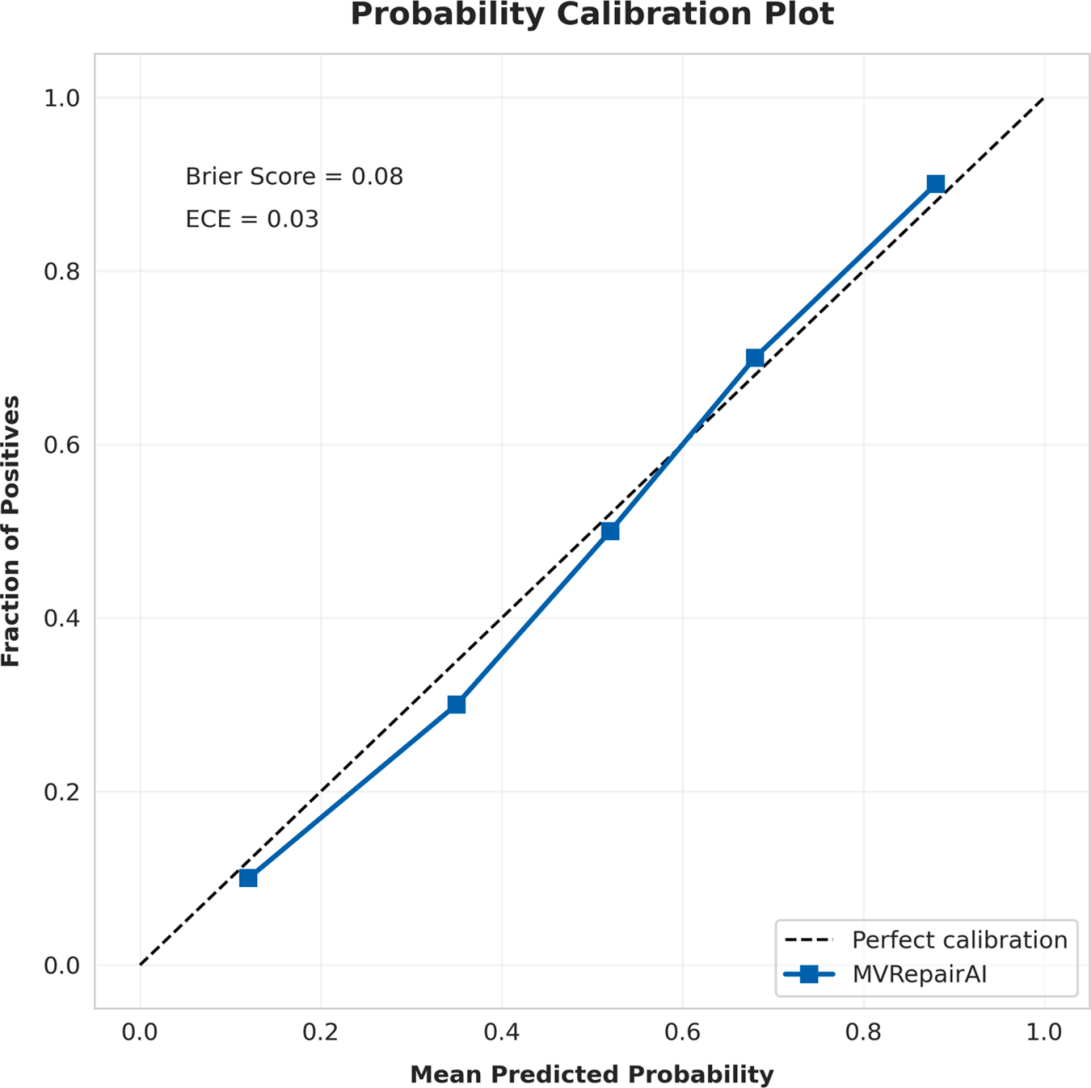
Calibration of predictive probabilities: a probability calibration plot. The plot compares predicted versus actual event probabilities. It includes the Brier score and expected calibration error (*ECE*) and is essential for evaluating probabilistic predictions. *MVRepairAI*, Mitral Valve Repair Artificial Intelligence.

SHapley Additive exPlanations analysis was used to measure prolapse location (relative importance: 0.18), annular dilatation (0.15), and left ventricular ejection fraction (0.12) as overarching predictors (Figure 4). Probability calibration affirmed outstanding reliability (Brier score 0.08; expected calibration error 0.03; Figure E8).

**Figure E9:**
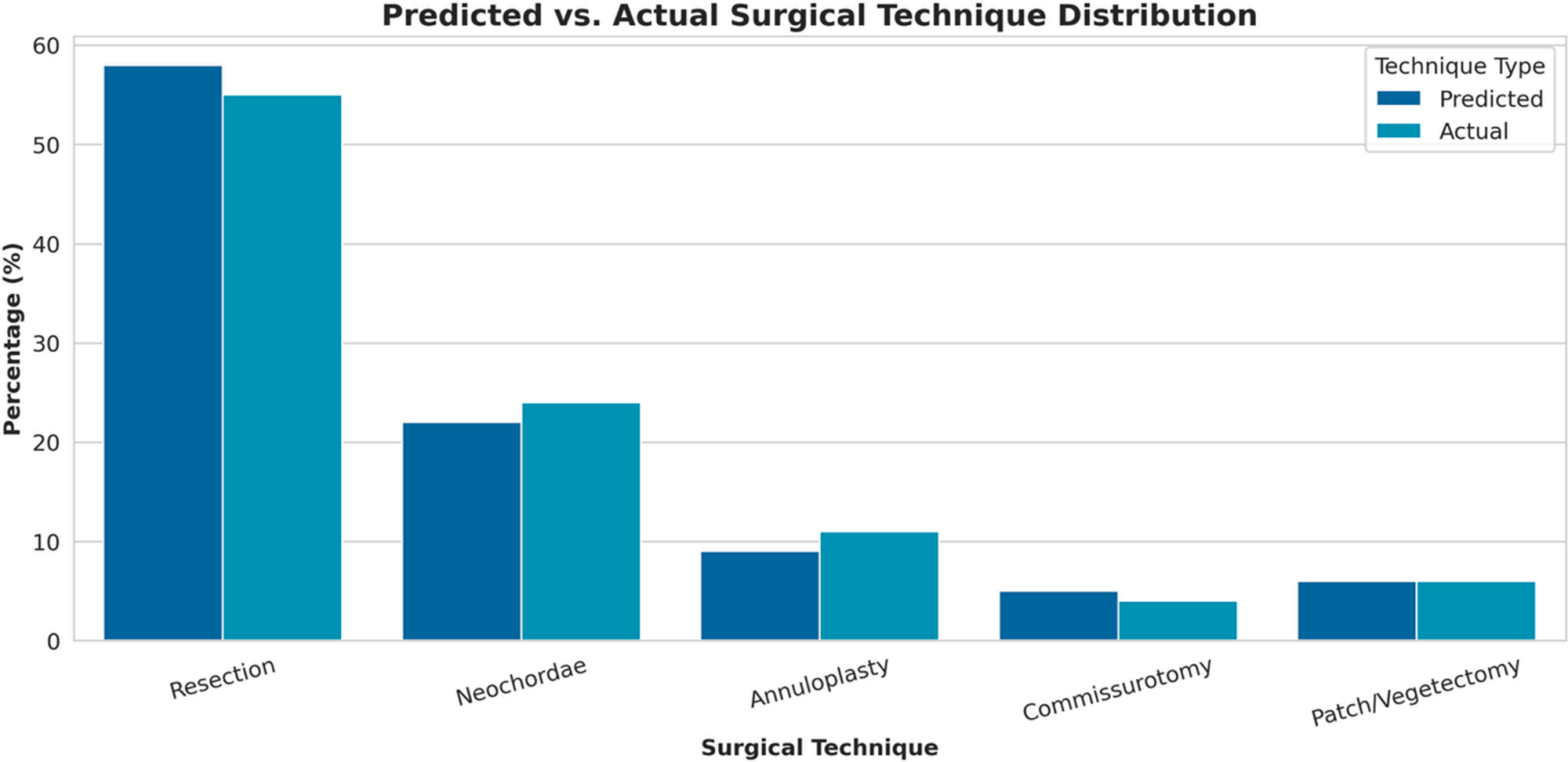
Predicted versus actual surgical technique distribution. Close alignment between model-predicted and actual techniques is shown.

**Figure E10:**
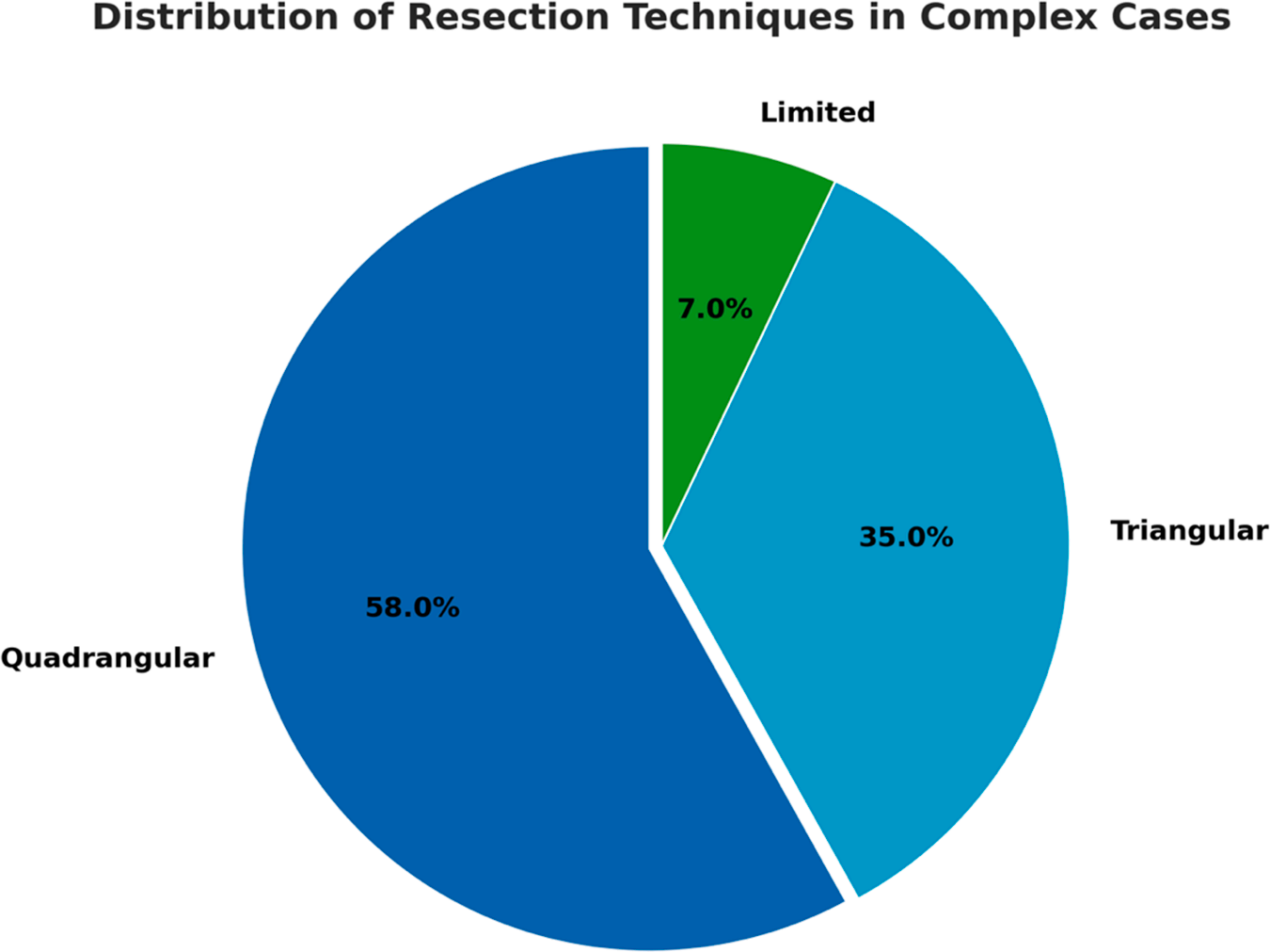
Distribution of resection techniques in complex cases. Quadrangular resection was shown to be the preference in complex repairs.

Baseline echocardiographic parameters differed significantly across etiologic strata, with functional mitral regurgitation characterized by larger ventricular dimensions and lower ejection fraction, and degenerative disease by higher effective regurgitant orifice area and regurgitant volume (Table E8). The distribution of primary repair techniques (Table E9) was dominated by resection and neochordal implantation in degenerative disease, with annuloplasty-only repair used almost exclusively for functional mitral regurgitation. Model-predicted technique frequencies closely mirrored the observed operative distribution across all technique categories (Figure E9), and among high-complexity valves quadrangular resection predominated (58%) over triangular (35%) and limited (7%) resection (Figure E10).

## Discussion

The development of MVRepairAI fills a crucial gap in MV surgical planning by using a structured algorithmic approach that converts preoperative imaging into operative strategies. Unlike existing models such as European System for Cardiac Operative Risk Evaluation or the Society of Thoracic Surgeons, which only report mortality risk without offering procedural guidance, or “black box” ML that predicts outcomes without integrating surgical logic, the MVRepairAI framework incorporates Carpentier's functional taxonomy to provide precise technical recommendations.^24^, ^25^, ^26^, ^27^ It achieved a 92.2% match with intraoperative decisions, outperforming automated annular quantification tools like eSie Valves and transcatheter intervention planners like MitraClip simulators, which lack adaptability to varying anatomies.^28^^,^^29^ By focusing on anatomical detail and surgical adaptation, MVRepairAI effectively bridges the well-known gap between diagnosis and surgery.^30^

The model is conceptualized as an augmented intelligence tool, designed to assist rather than replace surgical professionals. For an individual surgeon, it functions as an efficient, data-driven checklist that ensures all relevant anatomical features are considered. A multidisciplinary team can use it as a standardized anatomical evaluation tool, facilitating and normalizing discussions by minimizing interpretative discrepancies. Furthermore, the entire field benefits from this model by providing a clear and foundational framework that connects preoperative anatomy with surgical techniques in future outcome studies, thereby identifying which strategies yield the most favorable results over time.

The algorithm's hierarchical structure is meticulously designed to mimic a surgeon's cognitive processes, facilitating a smooth integration of computational logic with clinical expertise. Within this framework, the repair of commissural pathology is prioritized as the principal feature, aligning with the well-established principles of valve hemodynamics.^24^ Furthermore, SHapley Additive exPlanations–interpretable predictions elucidate the significant influence of prolapse location and annular dilatation on determining the appropriate treatment, offering more transparency than traditional black-box neural networks.^31^ This stands in stark contrast to physics-based finite element models, which simulate device effects but demand excessive computational resources for intraoperative use.^32^ However, the rule-based system is inherently limited in addressing unencoded anatomical variants, a limitation shared by other clinically interpretable AI systems in cardiac surgery.^9^

### Research Imperatives and Limitations

A number of limitations should be rigorously acknowledged. Because institutional protocols at tertiary centers might not accurately reflect broader practice patterns, the single-center retrospective design naturally restricts generalizability. Given the worldwide prevalence of rheumatic heart disease, the underrepresentation of ischemic (n = 6) and rheumatic (n = 1) etiologies limits the pathologic scope. Furthermore, the predictions by the model are essentially conditioned on how different clinicians have interpreted preoperative echocardiograms and documented them in the clinical reports. Variability in the way different echocardiographers describe segmental pathology and score complexity is likely to affect the consistency of such input data. Next iterations might avoid this by incorporating quantitative, algorithm-based analyses of original echocardiographic images (eg, automated leaflet tracking, 3-dimensional volumetric assessment) to derive more objective and reproducible input features.

Moreover, neglecting the use of intraoperative TEE disregards a vital component in the decision-making process for complex repairs. Most notably, the lack of longitudinal durability data impedes the assessment of the algorithm's ultimate clinical metric: the long-term success of repairs. These limitations highlight essential research priorities. A multicenter validation across diverse health care settings is imperative to evaluate performance in environments with varying resources. The integration of real-time, 3-dimensional echocardiography with hemodynamic data may uncover dynamic intraoperative nuances. The clinical impact would be substantiated by a long-term study examining the correlation between algorithmic recommendations and a decade of remission. Hybrid architectures that combine rule-based logic with supervised learning could enhance adaptability for exceptional anatomical configurations.

### Contextualizing Clinical Innovation

MVRepairAI exemplifies “augmented intelligence” in the field of cardiac surgery, where the technology is used to enhance rather than replace surgical expertise.^33^ By transitioning from preoperative imaging to guiding maps, it addresses the long-standing variability in surgical outcomes that is contingent upon the surgeon's level of experience.^34^

Future iterations that incorporate biomechanical simulations of leaflet stress may facilitate the selection of techniques aimed at enhancing durability,^35^ whereas integration into transcatheter planning ecosystems could harmonize open and minimally invasive strategies.^29^ As ML continues to transform cardiac interventions,^33^ this framework lays the basis for making accessible high-quality valve repair through standardized, patient-specific restoration logic.

The integration of MVRepairAI into the clinical workflow could be effectively realized through its incorporation into electronic health records or a specialized surgical planning platform. Before surgery, the system may autonomously analyze structured echocardiogram data to generate a preoperative diagnostic report, which outlines the most probable successful strategy for the repair. The surgeon, retaining ultimate decision-making authority, can use this report as a reference tool during multidisciplinary team meetings, where it is standardized by anatomical considerations. In the operating room, an instrument panel may feature a simplified version that enables the surgeon to compare the preoperative plan with the ongoing operation in real time, particularly if this dashboard is integrated with the versions intended for intraoperative imaging.

## Conclusions

MVRepairAI represents a significant advancement in mitral surgery, demonstrating the potential of AI to convert preoperative imaging into surgically pertinent plans for anatomical and functional classification. Although its hierarchical structure shows substantial concordance with operative approaches across diverse pathologic presentations, the review acknowledges limitations in generalizability as a result of the single-center retrospective design and the underrepresentation of certain etiologies.

The lack of integration of intraoperative real-time data and durability metrics over time further highlights current application limitations. Future refinements require rigorous multicenter validation, integration of dynamic hemodynamic evaluations, and longitudinal outcomes analysis. Despite these limitations, this platform serves as a foundational template for standardized, patient-specific valve analysis, a step toward synergizing computational logic with surgical expertise, with the potential to contribute to more consistent and high-quality repair strategies upon future validation.

## Conflict of Interest Statement

The authors reported no conflicts of interest.

The *Journal* policy requires editors and reviewers to disclose conflicts of interest and to decline handling or reviewing manuscripts for which they may have a conflict of interest. The editors and reviewers of this article have no conflicts of interest.

## Figures and Tables

**Table E1:** Etiological classification system for mitral valve pathology

Class	Carpentier^24^ type	Key echocardiographic features	Mechanism of regurgitation	Clinical implications
Degenerative	II	Isolated segment prolapse (typically P2), thin mobile leaflets, no calcification	Excessive leaflet motion	Standard repair techniques (resection/neochordae) highly effective
Barlow	II	Bileaflet prolapse, leaflet thickening (>5 mm), excess tissue, annular dilatation	Myxomatous degeneration	Requires extensive resection with sliding plasty/annular plication, large annuloplasty rings
Functional	IIIb	Restricted leaflet motion, annular dilatation, normal leaflet morphology	Ventricular distortion	Annuloplasty alone often insufficient; may require subvalvular apparatus modification
Rheumatic	IIIa	Commissural fusion, leaflet thickening, restricted motion, diastolic doming	Fibrotic retraction	Commissurotomy essential; patch augmentation often required
Endocarditis	I/II/III	Vegetations, leaflet perforations, annular abscesses	Tissue destruction	Radical debridement needed; high likelihood of patch repair
Ischemic	IIIb	Regional wall motion abnormalities, asymmetric tethering (often P3), annular dilatation	Papillary muscle displacement	Ventricular restoration critical alongside annuloplasty
Mixed	Variable	Combination of prolapse (II) and restriction (III) features	Multiple mechanisms	Hybrid repair strategies required

**Table E2:** MVCS components and weighting

Component	Subcomponent	Weight	Definition
Prolapse	Posterior segment (P1, P2, P3)	1 per segment	Systolic displacement >2 mm beyond annular plane
	Anterior segment (A1, A2, A3)	2 per segment	Systolic displacement >2 mm beyond annular plane
	Commissure (anterolateral/posteromedial)	2 per commissure	Prolapse involving commissural zones
Leaflet restriction	Any segment	2	Restricted motion (Carpentier type III)
Calcification	Leaflet/chordae/papillary (no annular)	2	Echogenic foci with shadowing on nonannular structures
	Annular involvement	3	Calcium extending into annular tissue
Previous mitral repair	History of repair	3	Documented previous mitral valve repair surgery

Mitral valve complexity score (MVCS) calculation system is shown. Based on Anyanwu and colleagues.^E1^

**Table E3:** Complexity stratification

Total score	Complexity group	Clinical interpretation
1	Simple	Isolated posterior prolapse (typically P2); standard resection/annuloplasty sufficient
2-4	Intermediate	Multisegment posterior or anterior involvement; requires advanced techniques (eg, neochordae)
≥5	Complex	Bileaflet/multisegment prolapse + ancillary factors (calcification, restriction, or re-repair)

Example calculations:

**Table:** 

Clinical scenario	Components	Calculation	Total score	Group
Isolated P2 prolapse	P2 (1)	1	1	Simple
A2 prolapse + mild leaflet calcification	A2 (2) + leaflet calcification (2)	2 + 2	4	Intermediate
Barlow's disease: P2/P3/A2/A3 prolapse + annular calcification	P2 (1) + P3 (1) + A2 (2) + A3 (2) + annular calcification (3)	1 + 1 + 2 + 2 + 3	9	Complex
Anterior commissure prolapse + previous repair	Anterior commissure (2) + previous repair (3)	2 + 3	5	Complex
P1/A1 prolapse + leaflet restriction	P1 (1) + A1 (2) + leaflet restriction (2)	1 + 2 + 2	5	Complex

Mitral valve complexity score (MVCS) calculation system is shown. Based on Anyanwu and colleagues.^E1^

**Table E4:** Patient demographic and clinical characteristics

Characteristic	Overall (N = 180)	Degenerative (n = 143)	Functional (n = 11)	Endocarditis (n = 9)
Age, y	67.2 ± 9.8	68.1 ± 8.9	71.3 ± 7.5	59.4 ± 11.2
Female sex	78 (43.3%)	62 (43.4%)	5 (45.5%)	3 (33.3%)
BMI, kg/m²	26.4 ± 4.1	26.1 ± 3.9	29.2 ± 4.8	25.3 ± 3.5
LVEF, %	55.3 ± 8.2	56.1 ± 7.3	42.7 ± 6.9	53.8 ± 8.1
NYHA class III/IV	123 (68.3%)	97 (67.8%)	9 (81.8%)	6 (66.7%)
Complexity score	3.4 ± 1.8	3.8 ± 1.6	1.2 ± 0.9	4.1 ± 1.9
High complexity (score ≥4)	63 (35.0%)	58 (40.6%)	0 (0%)	5 (55.6%)
EuroSCORE II	3.2 ± 4.1	2.8 ± 3.2	10.5 ± 8.7	4.9 ± 5.6

Only the principal etiologic subgroups are shown separately. The full cohort (N = 180) additionally comprises ischemic (n = 6), rheumatic (n = 1), and mixed/other (n = 10) cases; mixed/other cases could not be assigned to a single etiologic stratum. *BMI*, Body mass index; *LVEF*, left ventricular ejection fraction; *NYHA*, New York Heart Association; *EuroSCORE II*, European System for Cardiac Operative Risk Evaluation.

**Table E5:** Model performance metrics

Metric	Value	95% CI	*P* value
Overall accuracy	92.22%	89.1%-94.7%	<.001
Precision	89.5%	86.2%-92.1%	<.001
Recall	91.7%	88.8%-94.0%	<.001
F1 score	90.6%	87.6%-92.9%	<.001
Resection type accuracy	94.3%	91.5%-96.3%	<.001
Cohen kappa	0.89	0.85%-0.93	<.001

*MVRepairAI*, Mitral Valve Repair Artificial Intelligence.

**Table E6:** Etiology-based performance

Etiology	Cases (n)	Accuracy (95% CI)	Precision (95% CI)	Key surgical technique
Degenerative	143	95.1% (92.3%-97.9%)	93.2% (90.1%-96.3%)	Segment-specific resection
Functional	11	95.5% (89.1%-100%)	94.1% (87.2%-100%)	Annuloplasty only
Endocarditis	9	88.9% (78.3%-99.5%)	85.3% (73.1%-97.5%)	Pericardial patch repair
Ischemic	6	91.7% (81.5%-100%)	89.2% (78.1%-100%)	Annuloplasty + chordal repair
Rheumatic	1	100% (100%-100%)	100% (100%-100%)	Commissurotomy

**Table E7:** Subgroup analysis by complexity class

Complexity class	Cases, n	Accuracy (95% CI)	Resection accuracy (95% CI)	Common techniques
High (score ≥4)	63	90.5% (86.2%-94.8%)	94.1% (90.3%-97.9%)	Quadrangular resection (87%)
Moderate (score 2-3)	89	93.3% (90.1%-96.5%)	92.6% (88.7%-96.5%)	Triangular resection (76%)
Low (score <2)	28	92.9% (87.3%-98.5%)	N/A	Neochordae/annuloplasty (96%)

*N/A*, Not available.

**Table E8:** Preoperative echocardiographic characteristics

Parameter	Overall (N = 180)	Degenerative (n = 143)	Functional (n = 11)	Endocarditis (n = 9)	*P* value
LVEDD, mm	62.3 ± 7.8	59.8 ± 6.2	68.9 ± 8.1	61.5 ± 7.3	<.001
LVESD, mm	41.5 ± 6.9	39.2 ± 5.8	48.3 ± 7.5	40.1 ± 6.1	<.001
LVEF, %	55.3 ± 8.2	56.1 ± 7.3	42.7 ± 6.9	53.8 ± 8.1	<.001
sPAP, mm Hg	45.2 ± 12.3	42.8 ± 10.9	58.3 ± 14.2	47.1 ± 11.8	<.001
EROA, mm²	45.8 ± 18.3	51.2 ± 16.7	28.9 ± 9.8	39.7 ± 12.4	<.001
Regurgitant volume, mL	68.5 ± 25.1	73.9 ± 23.8	42.3 ± 14.2	59.8 ± 20.3	<.001
Vena contracta, mm	7.2 ± 2.1	7.8 ± 1.9	5.1 ± 1.2	6.9 ± 2.0	<.001
Annular diameter, mm	38.7 ± 4.8	39.5 ± 4.2	42.1 ± 5.3	36.8 ± 3.9	.003

Only the principal etiologic subgroups are shown separately. The full cohort (N = 180) additionally comprises ischemic (n = 6), rheumatic (n = 1), and mixed/other (n = 10) cases; mixed/other cases could not be assigned to a single etiologic stratum. *LVEDD*, Left ventricular end-diastolic volume; *LVESD*, left ventricular end-systolic volume; *sPAP*, systolic pulmonary artery pressure; *EROA*, effective regurgitant orifice area.

**Table E9:** Surgical technique distribution

Technique	Overall (N = 180)	Degenerative (n = 143)	Functional (n = 11)	Endocarditis (n = 9)	Complexity class
Quadrangular resection	57 (31.7%)	55 (38.5%)	0 (0%)	2 (22.2%)	High (94.7%)
Triangular resection	53 (29.4%)	50 (35.0%)	1 (9.1%)	2 (22.2%)	Moderate (85.2%)
Neochordae	44 (24.4%)	38 (26.6%)	1 (9.1%)	5 (55.6%)	High (61.4%)
Annuloplasty only	17 (9.4%)	0 (0%)	9 (81.8%)	0 (0%)	Low (100%)
Commissurotomy	11 (6.1%)	11 (7.7%)	0 (0%)	0 (0%)	High (100%)
Pericardial patch	9 (5.0%)	0 (0%)	0 (0%)	9 (100%)	High (100%)
Edge-to-edge repair	6 (3.3%)	6 (4.2%)	0 (0%)	0 (0%)	Moderate (100%)

Only the principal etiologic subgroups are shown separately. The full cohort (N = 180) additionally comprises ischemic (n = 6), rheumatic (n = 1), and mixed/other (n = 10) cases; mixed/other cases could not be assigned to a single etiologic stratum.
